# Selection of *Bacillus* spp. for Cellulase and Xylanase Production as Direct-Fed Microbials to Reduce Digesta Viscosity and *Clostridium perfringens* Proliferation Using an *in vitro* Digestive Model in Different Poultry Diets

**DOI:** 10.3389/fvets.2015.00025

**Published:** 2015-08-17

**Authors:** Juan D. Latorre, Xochitl Hernandez-Velasco, Vivek A. Kuttappan, Ross E. Wolfenden, Jose L. Vicente, Amanda D. Wolfenden, Lisa R. Bielke, Omar F. Prado-Rebolledo, Eduardo Morales, Billy M. Hargis, Guillermo Tellez

**Affiliations:** ^1^Department of Poultry Science, University of Arkansas, Fayetteville, AR, USA; ^2^Departamento de Medicina y Zootecnia de Aves, Facultad de Medicina Veterinaria y Zootecnia, Universidad Nacional Autonoma de Mexico, Mexico City, Mexico; ^3^Pacific Vet Group-USA, Inc., Fayetteville, AR, USA; ^4^Facultad de Medicina Veterinaria y Zootecnia, Universidad de Colima, Colima, Mexico; ^5^Departamento de Produccion Agricola y Animal, Universidad Autonoma Metropolitana, Mexico City, Mexico

**Keywords:** *Clostridium perfringens*, *Bacillus*-DFM, spore, enzymes, viscosity

## Abstract

Previously, our laboratory has screened and identified *Bacillus* spp. isolates as direct-fed microbials (DFM). The purpose of the present study was to evaluate the cellulase and xylanase production of these isolates and select the most appropriate *Bacillus* spp. candidates for DFM. Furthermore, an *in vitro* digestive model, simulating different compartments of the gastrointestinal tract, was used to determine the effect of these selected candidates on digesta viscosity and *Clostridium perfringens* proliferation in different poultry diets. Production of cellulase and xylanase were based on their relative enzyme activity. Analysis of 16S rRNA sequence classified two strains as *Bacillus amyloliquefaciens* and one of the strains as *Bacillus subtilis*. The DFM was included at a concentration of 10^8^ spores/g of feed in five different sterile soybean-based diets containing corn, wheat, rye, barley, or oat. After digestion time, supernatants from different diets were collected to measure viscosity, and *C. perfringens* proliferation. Additionally, from each *in vitro* simulated compartment, samples were taken to enumerate viable *Bacillus* spores using a plate count method after heat-treatment. Significant (*P* < 0.05) DFM-associated reductions in supernatant viscosity and *C. perfringens* proliferation were observed for all non-corn diets. These results suggest that antinutritional factors, such as non-starch polysaccharides from different cereals, can enhance viscosity and *C. perfringens* growth. Remarkably, dietary inclusion of the DFM that produce cellulase and xylanase reduced both viscosity and *C. perfringens* proliferation compared with control diets. Regardless of diet composition, 90% of the DFM spores germinated during the first 30 min in the crop compartment of the digestion model, followed by a noteworthy increased in the intestine compartment by ~2log_10_, suggesting a full-life cycle development. Further studies to evaluate *in vivo* necrotic enteritis effects are in progress.

## Introduction

Necrotic enteritis (NE) in broilers is a multi-factorial disease with severe economic implications (1). It is caused by type A strains of *Clostridium perfringens* that are specific to poultry with toxin types alpha and NetB (2, 3). Coccidia infections are the most common pre-requisite for NE to occur (4), however, dysbacteriosis associated with diet ingredients, changes in feed ration, immunosuppression, *Salmonella* infections, and/or removal of the use of quimioterapeutics are known to predispose birds to NE (1–5). Antibiotic growth promoters (AGPs) are commonly used to mitigate the incidence of enteric diseases, such as NE. Nevertheless, concerns regarding the development of antibiotic-resistant microorganisms and social pressures have led to a tendency to ban AGPs in poultry production (6). In this scenario, there is an imperative necessity to find feasible alternatives for AGPs to maintain poultry health (7). In fact, the use of selected strains of various beneficial microorganisms from the genus *Bacillus* and *Lactobacillus* have shown to be a suitable option for the poultry industry (8). *Bacillus* spp. are Gram-positive, aerobe, motile, and usually found in soil and water sources, as well as in the gastrointestinal tract of animals and humans (9). Different *Bacillus* spp. have already been studied and extensively used as a source of industrial enzymes as well as antibiotics by biotechnology companies (10). However, the production of most of these enzymes depends on the intense metabolic changes associated with environmental conditions (11–13). During extreme environmental conditions, vegetative cells of *Bacillus* spp. form endospores, which are considered, the toughest way of life on Earth (14).

The use of spores from selected *Bacillus* strains, as direct-fed microbials (DFM), are shown to have the capacity to germinate and sporulate in the gastrointestinal tract of different animal species including poultry. Thus, they become metabolically active *in vivo*, imparting numerous nutritional benefits including the production of extracellular enzymes, such as protease, lipase, cellulase, xylanase, phytase, and keratinase (15, 16), and other chemical compounds beneficial for the host (17).

In most of the USA and other countries, including Brazil, broiler feed is based primarily on corn and soybean meal. However, sometimes it is difficult to formulate least-cost diets using corn. Consequently, other cereals or ethanol by-products with variable concentrations of antinutritional factors are used as alternatives. When chickens are fed alternative grains with high levels of non-starch polysaccharides (NSP), an increase in digesta viscosity, poor nutrient digestibility, reduced bone mineralization, and occurrence of enteric diseases, such as NE, have been reported (18, 19). Hence, utilization of these feedstuffs in poultry diets usually result in decreased growth performance, intestinal dysbacteriosis, and detrimental litter conditions caused by sticky droppings (20, 21). For that reason, the inclusion of enzymes, such as carbohydrases, is a routine practice in poultry diets that contain grains with elevated NSP concentration values in comparison to corn (22, 23). However, there are inconveniences related to dietary inclusion of some enzymes, due to denaturation and lost of activity under high-pelletization temperatures commonly used in poultry rations. Therefore, the objective of the present study was to perform a selection of *Bacillus* spp. for cellulase and xylanase production as DFM, and evaluate them on digesta viscosity and *C. perfringens* proliferation in different poultry diets using an *in vitro* digestive model. The practical implication of the results will be to utilize cost-effective alternative grains in poultry feed formulation, and at the same time improve digestibility as well as production performance in birds using a more thermostable DFM product.

## Materials and Methods

### Diets

Five mash soybean-based broiler grower diets containing different cereals, such as corn, wheat, rye, barley, or oat, were used as substrate for bacterial growth during the *in vitro* digestive model. Experimental diets were formulated to approximate the nutritional requirements of broiler chickens as recommended by the NRC (24), and adjusted to breeder’s recommendations (25). No antibiotics, coccidiostats, or enzymes were added to the feed (Table 1). All diets were autoclaved and confirmed negative for *Bacillus* spp. spores. Later, these diets were inoculated with the respective spores (10^8^ spores/g of feed) of the *Bacillus*-DFM candidate according to various treatments.

**Table 1:** **Ingredient composition and nutrient content of different broiler chicken diets used for *in vitro* digestion with or without inclusion of *Bacillus*-DFM candidate spore on as-is basis^a^**.

Item	Corn-based diet	Wheat-based diet	Barley-based diet	Rye-based diet	Oat-based diet
**INGREDIENTS (g/kg)**					
Corn (80 g/kg CP)	619.6	–	–	–	–
Wheat (135 g/kg CP)	–	711.0	–	–	–
Barley (113 g/kg CP)	–	–	654.3	–	–
Rye (126 g/kg CP)	–	–	–	622.6	–
Oats (98 g/kg CP)	–	–	–	–	638.0
Soybean meal (475 g/kg CP)	298.2	203.9	241.9	264.6	260.0
Poultry oil	39.1	42.8	65.0	70.0	70.0
Dicalcium phosphate	16.9	17.1	17.0	16.6	16.4
Calcium carbonate	10.6	8.5	8.2	10.4	10.0
Salt	3.8	3.0	3.0	5.7	2.0
DL-Methionine	3.3	2.5	3.0	3.0	3.2
L-Lysine HCL	2.8	4.6	2.0	2.0	1.6
Threonine	1.2	2.1	1.1	0.6	0.6
Choline chloride 60%	2.0	2.0	2.0	2.0	2.0
Vitamin premix^b^	1.0	1.0	1.0	1.0	1.0
Mineral premix^c^	1.0	1.0	1.0	1.0	1.0
Antioxidant^d^	0.5	0.5	0.5	0.5	0.5
**CALCULATED ANALYSIS**					
Metabolizable energy (MJ/kg)	13.0	13.0	12.3	12.2	11.9
Crude protein (g/kg)	195.0	200.0	190.0	205.0	186.4

*^a^Inclusion of 10^8^ spore/g of feed mixed with calcium carbonate*.

*^b^Vitamin premix supplied per kilogram of diet: retinol, 6 mg; cholecalciferol, 150 μg; DL-α-tocopherol, 67.5 mg; menadione, 9 mg; thiamine, 3 mg; riboflavin, 12 mg; pantothenic acid, 18 mg; niacin, 60 mg; pyridoxine, 5 mg; folic acid, 2 mg; biotin, 0.3 mg; cyanocobalamin, 0.4 mg*.

*^c^Mineral premix supplied per kg of diet: Mn, 120 mg; Zn, 100 mg; Fe, 120 mg; copper, 10–15 mg; iodine, 0.7 mg; selenium, 0.2 mg; and cobalt, 0.2 mg*.

*^d^Ethoxyquin*.

### *In vitro* assessment of cellulase and xylanase production

Previous research conducted in our laboratory focused on isolation of several *Bacillus* spp. from environmental and poultry sources (26, 27). Isolates were then screened for production of cellulase and xylanase. For evaluation of cellulase activity, the cellulose-Congo red agar was used and consisted of 0.50 g of K_2_HPO_4_ (Fisher Scientific, San Francisco, CA, USA), 0.25 g of MgSO_4_ (Sigma Chemical Co, St. Louis, MO, USA), 1.88 g of ashed, acid-washed cellulose powder (J. T. Baker Chemical Inc, Phillipsburg, NJ, USA), 0.20 g of Congo red (J. T. Baker Chemical Inc, Phillipsburg, NJ, USA), 20 g of noble agar (Difco Laboratories, Detroit, MI, USA), and 1000 mL of distilled water (15). For evaluation of xylanase activity, the medium used to screen *Bacillus* isolates contained 3 g of NaNO_3_, 0.5 g of K_2_HPO_4_, 0.2 g of MgSO_4_⋅7H_2_O, 0.02 g of MnSO_4_⋅H_2_O, 0.02 g of FeSO_4_⋅H_2_O, 0.02 g of CaCl_2_⋅2H_2_O with 20 g of noble agar (Difco Laboratories, Detroit, MI, USA), and 1000 mL of distilled water. Besides, 1 g yeast extract and 5 g beechwood xylan (Sigma Chemical Co, St. Louis, MO, USA) were used as carbon sources (28). During the screening process, 10 μL of each *Bacillus* isolate were placed on the center of each plate containing cellulose or xylan media. After 24 h of incubation at 37°C, all plates were evaluated and the diameters of the zones of clearance were measured removing the diameter of the bacterial colony. The relative enzyme activity (REA) was calculated by using the formula: REA = diameter of zone of clearance divided by the diameter of the bacterial colony in millimeters. Based on REA test in each group, organisms were categorized in to excellent (REA > 5.0), good (REA > 2.0 to 5.0), or poor (REA < 2.0) REA (29). Each *Bacillus* strain was evaluated by triplicate, and the average measurements are presented in Table 2.

**Table 2:** **Relative enzyme activity values (REA) and *Clostridium perfringens* zone of inhibition produced by different *Bacillus* spp. strains present in the *Bacillus*-DFM candidate treatment**.

Measurements	AM1002	AM0938	JD17
**CELLULASE ACTIVITY AT 24 h**			
Colony size (mm)	5.7 ± 0.33^a^	6.0 ± 0.58^a^	6.3 ± 0.33^a^
Zone of clearance (mm)	35.2 ± 1.76^a^	30.7 ± 0.67^a,b^	29.3 ± 2.19^b^
REA^c^	6.2 ± 0.12^a^	5.1 ± 0.49^a,b^	4.7 ± 0.29^b^
**XYLANASE ACTIVITY AT 24 h**			
Colony size (mm)	5.0 ± 0.58^b^	6.7 ± 0.33^a,b^	7.3 ± 0.67^a^
Zone of clearance (mm)	31.7 ± 0.88^a^	32.0 ± 1.15^a^	29.0 ± 1.53^a^
REA^c^	6.3 ± 0.87^a^	4.9 ± 0.43^a,b^	4.0 ± 0.15^b^
***C. perfringens*** *AT 24 h*			
Zone of inhibition (mm)^d^	12.3 ± 1.45^a^	14.0 ± 1.00^a^	8.0 ± 1.15^b^

*^a,b^Superscripts within a row with no common superscript differ significantly *P* < 0.05*.

*^c^Relative enzyme activity values (REA) reflect the capacity to produce cellulase and xylanase enzymes by *Bacillus* spp. REA was calculated dividing the diameter of area of clearance by the diameter of the *Bacillus* colony. Based on REA test, organism can be categorized into three groups showing excellent (REA > 5.0), good (REA > 2.0–5.0), or poor (REA < 2.0) relative enzyme activity. All *Bacillus* spp. isolates were tested by triplicate. Data expressed as mean ± SE*.

*^d^Represents the diameter of the zone of inhibition observed at 24 h of incubation without the diameter of the bacterial colony. All *Bacillus* spp. isolates were tested by triplicate. Data expressed as mean ± SE*.

### DFM culture identification

Based on the REA results, three *Bacillus*-DFM candidates with excellent to good REA were selected. These candidates were then identified and characterized using a bioMerieux API 50 CHB test kit (bioMerieux, Marcy l’Etoile, FRA). Individual strain was also subjected to 16S rRNA sequence analysis to a specialized laboratory (Midi labs, Newark, DE, USA). Generally recognized as safe (GRAS) status of these three isolates was affirmed, as described by Wolfenden et al. (30). One of the three *Bacillus* strains (AM1002) was identified as *Bacillus subtilis*, and the other two isolates (AM0938 and JD17) were identified as *B. amyloliquefaciens* (Table 3). Following the identification, all three *Bacillus* candidate strains were sporulated and mixed in equal amounts during the *Bacillus*-DFM preparation process as described below and incorporated to the experimental diets.

**Table 3:** **Identification of *Bacillus* spp. isolates by bioMerieux API 50 CHB^a^ and 16S rRNA sequence analyses^b^ present in the *Bacillus*-DFM candidate treatment**.

Isolate	API50 CHB		16S rRNA sequence analysis	
	Taxon	% ID	Closest match	% ID
AM1002	*Bacillus subtilis/amyloliquefaciens*	99.2	*Bacillus subtilis*	100.0
AM0938	*Bacillus subtilis/amyloliquefaciens*	99.0	*Bacillus amyloliquefaciens*	99.7
JD17	*Bacillus subtilis/amyloliquefaciens*	99.4	*Bacillus amyloliquefaciens*	99.6

*^a^BioMerieux API 50 CHB test kit*.

*^b^16S rRNA sequence analysis*.

### Preparation of spore-based DFM

In an effort to grow high numbers of viable spores, modified version of a solid state fermentation media (SS) developed by Zhao et al. (31) was used. Briefly, to prepare the SS fermentation media, ammonia broth was added to a mixture of 70% rice straw and 30% wheat bran at the rate of 40% by weight. Then, the SS fermentation media was added to 250 mL Erlenmeyer flasks and sterilized by autoclaving for 30 min at 121°C. Each of the three *Bacillus* strains candidates was grown, individually, overnight at 37°C in test tubes containing 10 mL of tryptic soy broth (TSB, Becton Dickinson, Sparks, MD, USA). After incubation, 2 mL of each candidate culture were added separately to the previously prepared SS fermentation media flasks. The inoculated flasks were incubated for 24 h at 37°C to promote growth of the *Bacillus* spp. candidates, and incubated for another 72 h at 30°C to trigger the initiation of the sporulation process. Following this, the inoculated SS fermentation media was removed from the Erlenmeyer flasks, placed onto Petri dishes, and dried at 60°C for 18 h. Then, the SS fermentation media was aseptically ground into a fine powder that contained stable *Bacillus* spores (~10^11^ spores/g). One gram of spores from each isolate (1:1:1) was combined to produce the *Bacillus*-DFM candidate final product containing ~3 × 10^11^ spores/g. *Bacillus*-DFM candidate was included into each experimental diet to reach a final concentration of 10^8^ spores/g using a rotary mixer for 15 min. Samples of feed containing the DFM candidate were subjected to 100°C for 10 min to eliminate vegetative cells and validate the amount of spores per gram of feed after inclusion and mixing steps. Following heat-treatment, 10-fold dilutions of the same feed samples from the glass tubes were plated on tryptic soy agar plates (TSA, Becton Dickinson, Sparks, MD, USA); letting spores in the feed sample germinate to vegetative cells after incubation at 37°C for 24 h, hence representing the number of spores present per gram of feed.

### *Clostridium perfringens* strain

A strain of *C. perfringens* previously described in a NE challenge model was kindly donated by Dr. Jack. L. McReynolds, USDA-ARS, College Station, TX, USA (32). A frozen aliquot was shipped on ice to our laboratory and was amplified in TSB with sodium thioglycolate (Sigma-Aldrich, St Louis, MO, USA). The broth culture was plated on phenylethyl alcohol agar plates (PEA, Becton Dickinson, Sparks, MD, USA) with 5% sheep blood (Remel, Lenexa, KS, USA) to confirm purity, aliquots were made with 25% sterile glycerol and stored at −80°C until further use. A single aliquot was individually amplified in TSB with sodium thioglycolate overnight for the *in vitro* proliferation studies and the final dose was confirmed by plating 10-fold dilutions on TSA plates with sodium thioglycolate.

### *In vitro* assessment of antimicrobial activity against *Clostridium perfringens*

The three *Bacillus* isolates present in the *Bacillus*-DFM candidate treatment were individually cultured aerobically overnight on TSA and screened for *in vitro* antimicrobial activity against *C. perfringens* as reported previously (33). Briefly, 10 μL of each *Bacillus* isolate were placed on the center of TSA plates, and incubated for 24 h at 37°C. Then, the plates with visible *Bacillus* colonies were overlaid with TSA with sodium thioglycolate containing 10^6^ cfu/mL of *C. perfringens*, and all plates were incubated anaerobically at 37°C. After 24 h of incubation, all plates were evaluated and the diameters of the zones of inhibition were measured removing the diameter of the bacterial colony. Each *Bacillus* strain was evaluated by triplicate, and the average measurements of antimicrobial activity against *C. perfringens* are presented in Table 2.

### *In vitro* digestion assay

The *in vitro* digestion model used in the present study was based on previous publications, with minor modifications (34, 35), and the assay was performed with five different experimental diets, with or without *Bacillus*-DFM candidate, in quintuplicates. Briefly, for all the gastrointestinal compartments simulated during the *in vitro* digestion model, a biochemical oxygen demand incubator (VWR, Houston, TX, USA) set at 40°C (to simulate poultry body temperature), customized with an standard orbital shaker (19 rpm; VWR, Houston, TX, USA) was used for mixing the feed content. Additionally, all tube samples were held at an angle of 30° inclination to facilitate proper blending of feed particles and the enzyme solutions in the tube. The first gastrointestinal compartment simulated was the crop, where 5 g of feed and 10 ml of 0.03M hydrochloric acid (HCL, EMD Millipore Corporation, Billerica, MA, USA) were placed in 50 mL polypropylene centrifuge tubes and mixed vigorously reaching a pH value around 5.2. Tubes were then incubated for 30 min. Following this time, all tubes were removed from the incubator. To simulate the proventriculus as the next gastrointestinal compartment, 3000 U of pepsin per gram of feed (Sigma-Aldrich, St Louis, MO, USA) and 2.5 mL of 1.5M HCl were added to each tube to reach a pH of 1.4–2.0. All tubes were incubated for additional 45 min. The third and the final steps were intended to simulate the intestinal section of the gastrointestinal tract. For that, 6.84 mg of 8× pancreatin (Sigma-Aldrich, St Louis, MO, USA) in 6.5 mL of 1.0M sodium bicarbonate (Sigma-Aldrich, St Louis, MO, USA) were added, and the pH was adjusted to range between 6.4 and 6.8 with 1.0M sodium bicarbonate. All tube samples were further incubated for 2 h. Hence, the complete *in vitro* digestion process took 3 h and 15 min. After the digestion, supernatants from all the diets were obtained by centrifugation for 30 min at 2000 × *g*. All supernatants were then tested for viscosity and *C. perfringens* proliferation, as described below.

### Viscosity

Viscosity was measured using a Brookfield digital cone-plate viscometer fitted with a CP-40 spindle (Brookfield Engineering Laboratories Inc., Stoughton, MA, USA). From each supernatant, 0.5 mL was taken to measure viscosity at a shear rate of 42.5/s at 40°C to mimic body temperature of poultry. Viscosity was evaluated by quintuplicate per diet with or without inclusion of the *Bacillus*-DFM candidate and reported in centipoise (cP = 1/100 dyne s/cm^2^).

### *Clostridium perfringens* proliferation

Proliferation of *C. perfringens* was performed according to previously published methods (35), with minor modifications. A suspension of 10^5^ cfu/mL of *C. perfringens* was added to five replicates of each of the following groups: (1) 6 mL TSB with sodium thioglycolate as a positive control group; (2) 3 mL TSB with sodium thioglycolate plus 3 mL supernatant from each digested control non-DFM diet; (3) 3 mL TSB with sodium thioglycolate plus 3 mL supernatant from digested diets supplemented with *Bacillus*-DFM. Samples were incubated anaerobically at 40°C, with tubes set at 30° angle with constant shaking (200 rpm) for 4 h. After incubation, 10-fold serial dilutions were made from all treatment groups in 0.85% sterile saline. Then, 10 μL was plated on TSA with sodium thioglycolate and incubated for 24 h at 40°C, anaerobically. Results were expressed as log_10_ cfu of *C. perfringens*/mL.

### *In vitro* determination of spore persistence

Persistence of the *Bacillus*-DFM spores in the *in vitro* digestive model was also evaluated (five replicates per diet treatment). At each time point during the digestive simulation process (crop, proventriculus, and intestine), 0.2 mL was immediately loaded into 0.5 mL sterile centrifuge tubes and heat-treated (pasteurized) at 75°C for 10 min to eliminate the presence of vegetative cells (36). After pasteurization, samples were loaded into sterile 96-well flat bottom plate and 10-fold dilutions were made and plated on TSA. Plates were incubated for 24 h at 37°C on aerobic conditions to enumerate spores per gram of sample.

### Statistical analysis

Data from all measurements were subjected to One-way analysis of variance as a completely randomized design using the General Linear Models procedure of SAS (SAS version 9.1) (37). Means were separated with Duncan’s multiple-range test at *P* < 0.05 considered as significant. Data were reported as mean ± SE.

## Results

Isolates AM1002, AM0938, and JD17 were selected from a pooled of *Bacillus* isolates in our laboratory, based on the REA values for cellulose and xylanase, and the zone of inhibition for *C. perfringens* (Table 2). Isolate AM1002 showed a REA value of 6.2 and AM0938 showed a REA value of 5.1, both considered excellent REA values (>5.0) for cellulase activity (29); additionally, isolate JD17 showed a REA value of 4.7, which is considered good (>2.0–5.0) for cellulase production. A similar trend was observed for xylanase activity where isolate AM1002 showed a REA value of 6.3 (excellent); AM0938 showed a REA value of 4.8 (good), and isolate JD17 showed a REA value of 4.0 (good) for xylanase production. In the case of antimicrobial activity against *C. perfringens*, isolate AM0938 generated the largest diameter of the zone of inhibition with 14 mm, followed by isolates AM1002 and JD17 with 12 and 8 mm, respectively. Although enzyme production and antimicrobial activity were observed for all the isolates, individual differences were evident even in bacteria of the same species (Table 2). The API 50 CHB system characterized all three isolates as *B. subtilis/amyloliquefaciens* (Table 3). Analysis of 16S rRNA sequence classified two strains (AM0938, JD17) as *B. amyloliquefaciens* and one of the strains (AM1002) as *B. subtilis*, which was consistent with the results observed by the carbohydrate fermentation profile of the biochemical test.

The results of the evaluation of digesta viscosity of different diets with or without inclusion of a *Bacillus*-DFM candidate after *in vitro* digestion are summarized in Table 4. An evident increase in viscosity was observed in soybean-based diets containing wheat, barley, rye, and oats when compared to corn, being rye, and oat diets with the highest viscosity values. However, it was noteworthy to observe that dietary inclusion of the *Bacillus*-DFM candidate significantly (*P* < 0.05) reduced viscosity in all diets containing cereals different to corn in comparison to control diets without DFM inclusion (Table 4).

**Table 4:** **Evaluation of *in vitro* viscosity of different diets with or without inclusion of a *Bacillus*-DFM candidate**.

Diet	Viscosity (cP)^c^	
	Control	*Bacillus*-DFM
Corn-based	0.96 ± 0.01^a^	0.97 ± 0.01^a^
Wheat-based	1.55 ± 0.02^a^	1.28 ± 0.01^b^
Barley-based	1.75 ± 0.02^a^	1.34 ± 0.03^b^
Rye-based	8.40 ± 0.37^a^	2.39 ± 0.04^b^
Oat-based	36.9 ± 2.15^a^	1.34 ± 0.01^b^

*^a,b^Superscripts within a row with no common superscript differ significantly *P* < 0.05*.

*^c^Viscosity was measured after 3 h and 15 min of *in vitro* digestion at 40°C. Data expressed as mean ± SE*.

Table 5 summarizes the results of the proliferation of *C. perfringens* in the supernatant from different digested diets with or without inclusion of a *Bacillus*-DFM candidate. A significant increase in *C. perfringens* proliferation was observed in supernatants collected from control diets that contained wheat, barley, rye, and oat compared to the TSB positive control group. Startlingly, dietary inclusion of a *Bacillus*-DFM candidate in non-corn diets significantly reduced *C. perfringens* proliferation when compared to the control non-DFM supplemented diets. The corn-based diet showed similar cfu values of *C. perfringens* with or without inclusion of the *Bacillus*-DFM candidate.

**Table 5:** **Proliferation of *Clostridium perfringens*^d^ in different digested diets with or without inclusion of *Bacillus*-DFM candidate spore^e^**.

Diet^c^	TSB + Thio	Control diet	*Bacillus*-DFM
Corn-based	6.38 ± 0.13^a^	6.44 ± 0.19^a^	6.68 ± 0.08^a^
Wheat-based	6.12 ± 0.24^b^	7.12 ± 0.07^a^	5.20 ± 0.18^b^
Barley-based	6.36 ± 0.06^c^	7.50 ± 0.13^a^	6.86 ± 0.11^b^
Rye-based	6.05 ± 0.21^c^	7.15 ± 0.09^a^	6.68 ± 0.12^b^
Oat-based	6.12 ± 0.07^b^	6.96 ± 0.13^a^	5.76 ± 0.07^c^

*^a,b^Superscripts within a row with no common superscript differ significantly *P* < 0.05*.

*^c^Supernatant from each diet was used as part of the broth for *C. perfringens* growth. Data expressed as mean ± SE*.

*^d^Inoculum used 10^5^ cfu of *C. perfringens* and 10^8^ spores/g of *Bacillus*-DFM candidate*.

*^e^Data expressed in log_10_ cfu/mL*.

Persistence of the *Bacillus*-DFM candidate spores in the different gastrointestinal compartments simulated in the *in vitro* digestive model is presented in Table 6. Regardless of diet composition, on average, a reduction of more than half of a log_10_ was observed in the crop compartment during the first 30 min of incubation, and it was followed by a further significant ~2log_10_ reduction of spore counts in the proventriculus. Remarkably, in all diets, a significant increment in spore numbers, ~2log_10_ was observed during the final digestion step simulating intestinal conditions (Table 6).

**Table 6:** **Persistence of *Bacillus*-DFM candidate^c^ spore during *in vitro* digestion^f^ in different diets under variable biochemical conditions simulating different sections of the gastrointestinal tract of poultry^e^**.

Diet^d^	Crop (30 min)	Proventriculus (45 min)	Intestine (120 min)
Corn-based	7.32 ± 0.10^a^	5.43 ± 0.17^b^	7.20 ± 0.09^a^
Wheat-based	7.54 ± 0.06^a^	5.58 ± 0.10^b^	7.33 ± 0.19^a^
Barley-based	7.45 ± 0.16^a^	4.95 ± 0.21^b^	7.27 ± 0.08^a^
Rye-based	7.28 ± 0.10^a^	5.60 ± 0.22^b^	7.09 ± 0.17^a^
Oat-based	7.66 ± 0.07^a^	5.06 ± 0.15^b^	7.30 ± 0.15^a^

*^a,b^Superscripts within a row with no common superscript differ significantly *P* < 0.05*.

^c^Inclusion of 10^8^ spore/g of feed

*^d^Heat shock was induced by placing a sample of each simulated compartment in a water bath at 75°C for 10 min. Data expressed as mean ± SE*.

*^e^Data expressed in log_10_ cfu/mL*.

*^f^pH and time of incubation varied according to the simulated organ*.

## Discussion

High-energy diets have been utilized to maximize growth during starter, grower, and finisher phases of production. Consequently, the primary dietary energy sources in commercial broiler diets have been traditional cereal grains such as corn and sorghum. However, with the recent price volatility of common feed ingredients, the animal industry seeks alternative grains or industry by-products to include in diet formulations (38, 39). Wheat, barley, rye, and oat contain lower bioavailable energy, and elevated NSP levels in comparison to corn (40) are alternative options. However, these cereals have limited use in monogastric diets, because often high-inclusion results in relatively poor performance, detrimental litter conditions, and increase predisposition for NE (41–43). Hence, supplemental carbohydrases, such as NSP-degrading enzymes, have allowed to increase the utilization of these alternative ingredients by reducing their antinutritional effects (40, 44, 45). The carbohydrase market is accounted by two dominant enzymes: xylanases and cellulases. Other commercially available carbohydrases include α-amylase, α-galactosidase, β-glucanase, β-mannanase, and pectinase (46).

In the present study, the *Bacillus spp*. strains that conform the DFM candidate treatment were identified as either *B. subtilis* or *B. amyloliquefaciens* (Table 3), therefore being feasible for *in vivo* evaluation studies as they are GRAS candidates (12, 26–28). Furthermore, the three selected *Bacillus spp*. isolates showed a variable ability to produce cellulase and xylanase (Table 2), hence, in addition to the benefits that spores or vegetative cells can provide as probiotics (9, 12), they may improve the digestibility of cereals with high-soluble NSP (47).

The *Bacillus*-DFM candidate treatment also demonstrated effective antimicrobial properties against *C. perfringens*, which could be due to production of antimicrobial-like compounds and/or competition for nutrients (Tables 2 and 5). Little is known about the mechanisms underlying the higher incidence of NE in broilers fed diets containing cereals with elevated levels of NSP, but it could be related to a prolonged feed rate of passage and a reduction in the digestion of nutrients that later in the hind gut will be available for bacteria to growth (48). For *in vitro* evaluation of *C. perfringens* proliferation, TSB with sodium thioglycolate (positive control) groups were included. In the TSB group (positive control), the *C. perfringens* inoculum was increased ~0.5log_10_, after 4 h of incubation. However, it was interesting to observe a significant increase in *C. perfringens* proliferation in the supernatants collected from control non-DFM diets that contained wheat, barley, rye, and oat, compared with the enrichment TSB medium with sodium thioglycolate group (Table 5).

These results suggest that partial digestion of NSP grains and increased digesta viscosity provides a favorable nutritional environment that supports the growth of *C. perfringens*. Interestingly, dietary inclusion of a *Bacillus*-DFM candidate in non-corn diets significantly reduced both viscosity (Table 4) and *C. perfringens* proliferation (Table 5), when compared to control diets without DFM inclusion. This result shows the capacity of certain *Bacillus* isolates to inhibit the growth of pathogenic bacteria like *C. perfringens*, probably due to competition for nutrients, production of antimicrobial-like compounds, or changes in environmental conditions. Proliferation of *C. perfringens* in the corn-based diet remained constant with or without the inclusion of the *Bacillus*-DFM candidate and in the TSB positive control group (Table 5). This outcome could be related to the lower concentration of NSP usually found in corn grains in comparison to other cereals, which was also supported by low digesta viscosity values (Table 4). These results are in accordance with previous reports (35), however, it is important to mention that diet ingredients are just one of the multiple predisposing factors that could affect the incidence of NE in commercial conditions (49, 50).

Beneficial bacterial spores are popular as DFM, though little is known about their mode of action. Previous studies conducted in our laboratory, have demonstrated that ~90% of *Bacillus* spores of a selected strain germinate within 30 min under *in vitro* and *in vivo* model conditions, with relatively constant numbers of spores in each gastrointestinal compartment evaluated, hence, suggesting that full-life cycle may occurs (51). In the present study, regardless of the diet, similar *in vitro* persistence of the *Bacillus*-DFM candidate spores was observed in the different simulated compartments (Table 6). On average, a half log_10_ reduction in spore numbers were detected in the crop compartment suggesting spore germination. In the proventriculus compartment, a further ~2log_10_ reduction was shown, supporting our previous findings (27, 51), which suggest that further germination of spores occurs even at low pH environments. However, it was particularly interesting to observe a ~2log_10_ increment in spore counts in the intestinal simulated compartment (Table 6). The increment in the numbers of spores could be a response to bacterial metabolites, competition for oxygen and nutrients available, resulting in resporulation (17). The above observations also support previous reports suggesting that spore transiting through the gastrointestinal tract could potentially undergo a full-life cycle of germination and resporulation (36, 52). Moreover, it has been demonstrated that germination of spores into metabolically and functionally active vegetative cells, within a similar time frame, produced beneficial metabolic and immunological effects in different animal species (53–57).

In summary, our results confirm that poultry diets containing cereal grains with a higher content of NSP in comparison to corn can enhance viscosity and *C. perfringens* growth (18–21). Remarkably, the dietary inclusion of a selected *Bacillus*-DFM candidate in non-corn-based diets significantly reduced both viscosity and *C. perfringens* proliferation when compared with the control non-supplemented-diets. Additionally, *Bacillus*-DFM candidate spore persisted and change their amount according to the variable biochemical conditions of the *in vitro* digestive model; therefore, supporting the hypothesis of the possible full-life cycle development in the gastrointestinal tract. The results from the present *in vitro* study encourage us to further evaluate the utilization of this *Bacillus*-DFM candidate in an *in vivo* NE model that we have developed in our laboratory (5), as well as to purify, characterize, and measure the international units of cellulase and xylanase that these *Bacillus* isolates produce. This knowledge will provide a valuable tool to use a stable DFM that produce exogenous enzymes in poultry diets.

## Conflict of Interest Statement

The authors declare that the research was conducted in the absence of any commercial or financial relationships that could be construed as a potential conflict of interest.
